# The therapeutic effect of PEI-Fe_3_O_4_/pYr-ads-8-5HRE-cfosp-IFNG albumin nanospheres combined with magnetic fluid hyperthermia on hepatoma

**DOI:** 10.3389/fonc.2023.1080519

**Published:** 2023-04-05

**Authors:** Hao Zhang, Suping Li, Fei Chen, Xingming Ma, Mingying Liu

**Affiliations:** ^1^ Department of Nuclear Medicine, Affiliated Hospital of North Sichuan Medical College, North Sichuan Medical College, Nanchong, China; ^2^ School of Health Management, Xihua University, Chengdu, China

**Keywords:** hyperthermia, combination therapy, magnetic nanoparticles, nano-drug, gene circuit

## Abstract

**Background:**

Hepatocellular carcinoma (HCC) is one of the most prevalent and deadly malignant tumors with serious clinical and socioeconomic consequences. Although gene therapy holds great promise in the treatment of hepatoma, its clinical applications are hindered by uncontrolled gene transmission and transcription.

**Methods:**

The pY-ads-8-5HRE-cfosp-IFNG plasmid was constructed and identified by double enzyme digestion and gene sequencing. The expression of pYr-ads-8-5HRE-cfosp-IFNG in HepG2 cells was detected by quantitative PCR. PEI-Fe3O4/pYr-ads-8-5HRE-cfosp-IFNG albumin nanospheres were prepared and characterized. In vitro heating test of magnetic albumin nanospheres in an alternating magnetic field (AMF) was carried out. The therapeutic effect of PEI-Fe3O4/pYr-ads-8-5HRE-cfosp-IFNG albumin nanospheres on hepatocellular carcinoma was investigated by cell and animal experiments. After treatment, mice blood was collected for clinical biochemical analysis and histopathological evaluation of major organs was performed to assess potential adverse effects of treatment.

**Results:**

Double enzyme digestion and gene sequencing showed that the pY-ads-8-5HRE-cfosp-IFNG plasmid was constructed successfully. QPCR results showed that the IFNγ transcript level in the PEI-Fe3O4/pYr-ads-8-5HRE-cfosp-IFNG group was higher than that in the PEI-Fe3O4/pYr-ads-8-cfosp-IFNG group after being treated with hypoxia (P<0.05). TEM revealed that the self-prepared PEI-Fe3O4/pYr-ads-8-5HRE-cfosp-IFNG albumin nanospheres exhibit an approximately spherical or elliptical shape. The hydrodynamic size of the albumin nanospheres was 139.7 nm. The maximum temperature of 0.25 mg/mL solution is stable at about 44°C, which is suitable for tumor thermal therapy without damaging normal tissues. The relative cell inhibition rate of the radiation-gene therapy and MFH combination group was higher than that of other control groups in CCK8 experiment. (P<0.05) Flow cytometry showed that the apoptosis rate and necrosis rate of the combined treatment group were 42.32% and 35.73%, respectively, higher than those of the other groups. (P<0.05) In animal experiments, the mass and volume inhibition rates of the combined treatment group were 66.67% and 72.53%, respectively, higher than those of other control groups. (P<0.05) Clinical biochemical analysis and histopathological evaluation showed no abnormality.

**Conclusions:**

The results indicated the successful construction of the radiation-induced plasmid and demonstrated that the hypoxia enhancer could augment the expression of INFγ in a hypoxia environment. Gene therapy combined with magnetic fluid hyperthermia (MFH) has exhibited excellent outcomes in both cell and animal studies. Our experiments demonstrated that the PEI-Fe3O4/pYr-ads-8-5HRE-cfosp-IFNG albumin nanospheres system is a comprehensive treatment method for hepatoma, which can effectively combine immune genre therapy with hyperthermia.

## Introduction

In the ranking of malignant tumor incidence in the world, hepatoma is the fifth. It is also one of the malignant tumors with the highest morbidity and mortality in China. Hepatoma has the characteristics of insidious onset, rapid progression, early recurrence and poor prognosis (1). Many patients with hepatoma are diagnosed at an advanced stage. With the improvement of early diagnosis rate of hepatoma, the improvement of surgery and interventional therapy and the adhibition of comprehensive therapy, the prognosis of patients with early hepatoma is satisfactory. However, the prognosis of patients with middle to advanced hepatoma is still poor.

Hepatoma is a complex, multi-step biological process involving changes in adhesion factors, matrix metalloproteinases, corresponding signal transduction matrix, and related genes. With the deepening of research on molecular signaling pathways and the tumor microenvironment of hepatoma, gene therapy for hepatoma has rapidly developed into a new treatment mode after surgery, radiotherapy, chemotherapy, and interventional therapy.

Gene therapy is a kind of treatment method which uses molecular biology technology to transplant selected genes into patients to correct abnormal gene expression. Gene therapy is mainly applied to remedy illnesses that cause grievous damage to human health, such as infectious diseases, malignant tumors, cardiovascular illnesses, and genetic illnesses (2). At present, many gene therapy methods (such as suicide genes, immune genes, RNA interference, etc.) are increasingly used in tumor therapy and have achieved exciting results. IFNγ, IL-2, and TNFα are widely used in immune gene therapy, and their curative effects are clear. The local transcription and expression of immune genes in cancer cells can increase the immunogenicity of cancer cells, thus stimulating and enhancing the body’s immune response to cancer cells and eliminating them as soon as possible. After the cancer cells are killed, the expression of immune genes also stops, and the initial triggering effect is over. IFNγ kills tumors in two ways. On the one hand, it has a direct anti-tumor effect, which is realized by inducing differentiation and accelerating tumor cell apoptosis, restraining tumor cell proliferation (3, 4). After the cancer cells are killed, the expression of immune genes also stops, and the initial triggering effect is over. IFNγ kills tumors in two ways. On the one hand, it has a direct anti-tumor effect, which is realized by inducing differentiation and accelerating tumor cell apoptosis, restraining tumor cell proliferation (5). Immunogene therapy can be combined with the body’s immune response to treat tumors, making it a worthy research direction of gene therapy for hepatoma.

Radio-gene therapy is the fusion of therapeutic genes with radiation-induced promoters. Upon radiation/radionuclide exposure, the radiation promoter is activated, and the therapeutic gene is expressed. In this process, radiotherapy and gene therapy can synergistically kill tumors, and localization of anti-tumor gene expression can be achieved by local irradiation. Moreover, the dose of radiation in the combination therapy is smaller under the premise of achieving the same anti-tumor effect (6).

However, like traditional gene therapy, this method has some shortcomings, such as issues with specificity and safety. Additionally, the general hypoxic environment of solid tumors can reduce the efficiency of the promoter induced by radiation, which affects the curative effect and application of this therapy (7, 8). Some researchers have found that inserting the hypoxia-response element (HRE) sequence upstream of the gene promoter can effectively solve the aforementioned problems. HRE is an enhancer sequence that could enhance promoter sensitivity and activity in hypoxic environments (9, 10).

Naked DNA is unstable in organism because it is easily degraded by nuclease (11). So far, there are two main gene transfer carriers: viral systems and non-viral systems, each having its own strengths and weaknesses. Viral gene vectors are currently the most efficient ones, but they pose serious biosecurity risks and have some disadvantages, such as strong self-immunogenicity, poor target specificity, and limited gene capacity, which greatly limit their clinical application. Although non-viral vectors avoid significant safety risks, the transfection efficiency of most non-viral vectors is not satisfactory, making it difficult to obtain meaningful target gene expression (12). Nowadays, the most commonly used non-viral carrier transfection methods are liposome transfection and electroporation transfection. Both methods have high transfection efficiency. However, liposomes have high cytotoxicity, which causes them to be quickly cleared by the body. Therefore, the application of liposomes must first solve this problem. Electrotransfection can only be used *in vitro*, and the gene is only transiently expressed in cells or tissues, so it cannot be used for transfection *in vivo* (13). Therefore, solving the problem of gene transfer is still the primary challenge in current gene therapy.

In recent years, nanotechnology has received much attention in various fields, including its application in the development of gene transfer vectors (14–18). Nanoparticles-based gene transfer vectors have attracted the attention of scholars (19, 20) due to their low biotoxicity and large surface area (21–25). Compared with traditional vectors, nano-vectors offer several advantages, such as slow and sustained gene release and maintenance of effective concentration for an extended period of time. Moreover, nano-vectors are highly safe and can be repeatedly injected to improve transfection efficiency. Among the various types of magnetic nanoparticles (MNPs), Fe_3_O_4_ MNPs are the most frequently used due to their excellent biocompatibility, low immunogenicity, superparamagnetism, and other interesting properties (26). The superparamagnetism effect allows MNPs to target tissues under an applied magnetic field, achieving efficient transfection, which enables targeted gene therapy. Additionally, MNPs can be used for hyperthermia of tumors as they can be heated through magnetic induction in an external magnetic field.

Hyperthermia is a therapeutic method used to treat cancer by destroying cancer cells and making them more sensitive to chemotherapy and radiotherapy. Therefore, it is often used in combination with gene therapy, chemotherapy, and radiotherapy to produce synergistic and supplementary effects. However, achieving uniform heating of the tumor tissue to an ideal temperature without damaging normal tissue during treatment is a challenging technical problem. Magnetic fluid hyperthermia (MFH) is a tumor hyperthermia method that combines magnetic induction heating with nanotechnology. This treatment allows for targeted localization, meaning that only tissues with magnetic nanoparticles experience an increase in temperature under an external magnetic field, while tissues without magnetic nanoparticles are unaffected by heat. As the intake of magnetic nanoparticles in tumor tissues is much higher than that in normal tissues, MFH can destroy tumor tissues while simultaneously protecting normal tissues (27–29).

In some of our previous studies, we chemically prepared Fe_2_O_3_ and Fe_3_O_4_ magnetic nanoparticles and applied them to the hyperthermia of hepatoma and lung cancer with good results. The Curie temperature of the magnetic fluid can be controlled by adjusting the iron concentration. Fe_3_O_4_ nanoparticles can absorb electromagnetic waves below Curie temperature and heat up in alternating magnetic fields (AMF). Once the Curie temperature is reached, Fe_3_O_4_ nanoparticles becomes non-magnetic, loses their capacity to take in electromagnetic waves, and the temperature begins to drop. When the temperature is below the Curie temperature, the Fe_3_O_4_ magnetic fluid begins to heat up again. Therefore, the temperature is cycled within the range set by Curie temperature consistently, such as 42-44 degrees Celsius, which is the effective hyperthermia temperature for cancer, and this temperature will not cause damage to normal tissues. Thus, we successfully solved the issue of temperature measurement and regulation in tumor hyperthermia, and improved the safety and stability of hyperthermia (23, 30).

Magnetic nanoparticles are coated with an albumin shell called magnetic albumin nanospheres (MANS) (31). Albumin nanospheres are often used as drug carriers, which can lower drug discharge rate and avoid drug inactivation in the course of transfer. Magnetic nanoparticles’ biocompatibility can be improved when albumin is coated with nano-materials. Placing a magnetic field outside the tumor site can make the drug-loaded magnetic albumin nanoparticles focus on tumor site. Thereby enhancing the anti-tumor effect of the antineoplastic drug and reducing the harm to normal tissues (32, 33).

Based on the above, we constructed a eukaryotic recombinant plasmid pYr-ads-8-5HRE-cfosp-IFNG and hypothesized that Fe_3_O_4_ nanoparticles could be used as gene carriers to target transport radiation-induced immune genes to tumor tissues in an external magnetic field. Meanwhile, Fe_3_O_4_ nanoparticles can also be used as magnetic media for hyperthermia, combining immunogene therapy and hyperthermia for tumor. Thus, we aim to explore an adjustable and precise combination therapy for hepatoma.

## Materials and methods

### Main materials

Restriction enzyme, Trizol, DNA maker and RNasin were purchased from Takara Biomedical Technology (Japan) Co., Ltd. Tryptone, and BSA were purchased from Sigma-Aldrich Co. (USA). HepG2 cells were purchased from the Institute of Biochemistry and Cell Biology, Shanghai Institute of Biological Sciences, Chinese Academy of Sciences. Plasmid extraction kit from Axygen (USA). Fetal bovine serum and DMEM medium were purchased from Gibco BRL Co. (USA); plasmid pDONR223-IFNG, pYr-ads-8-cfosp and pUC57-Simple-5HRE was synthesized by Biotech Co. Ltd., Changsha Ying Run (China).

### Construction and identification of pYr-ads-8-5HRE-cfosp-IFNG plasmid

**Figure 1:**
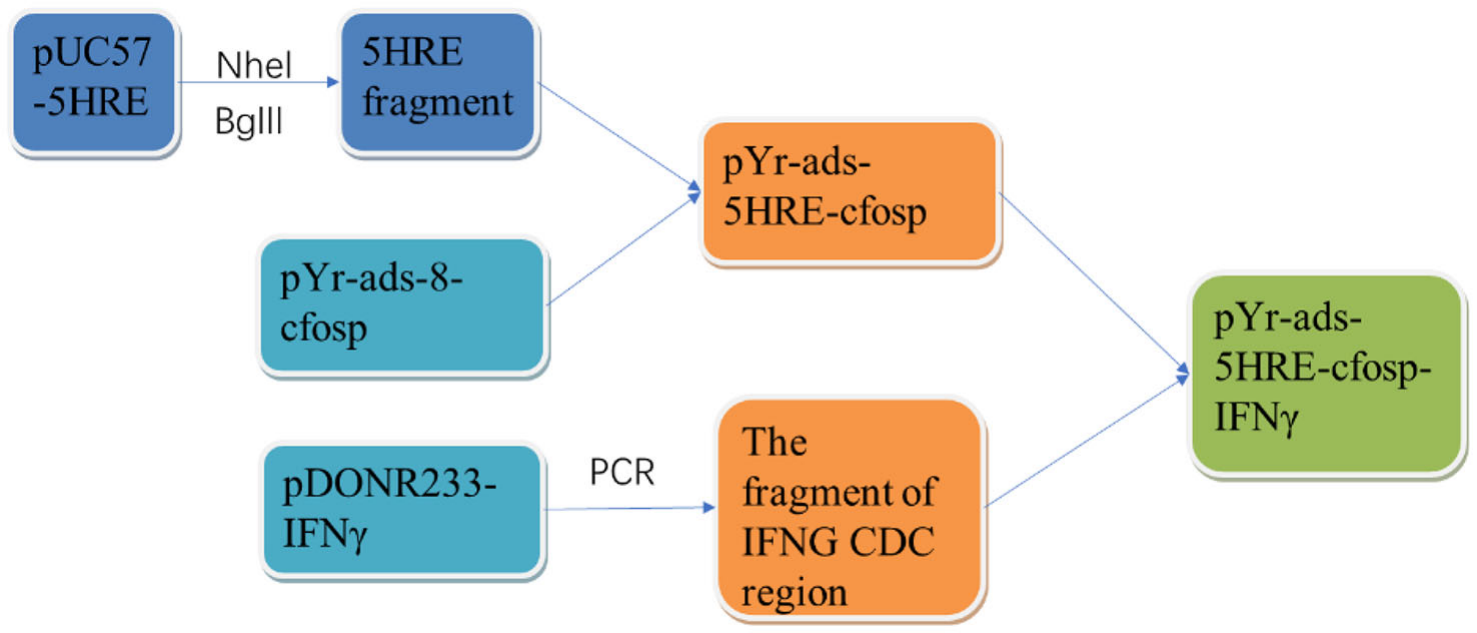
Flowchart of construction of pYr-ads-8-5HRE-cfosp-IFNG plasmid.

The 5HRE fragment was obtained by double restriction enzyme digestion of NheI and BglII, and the template plasmid was pUC57-5HRE plasmid. Then the 5HRE fragment was ligated with pYr-ads-8-cfosp to construct a plasmid containing 5HRE-cfosp fragment. With pDONR223-IFNG as template, the fragment of IFNG CDS region was amplified by PCR, and inserted into EcoRI-BamHI site of pYr-ads-8-5HRE-cfosp plasmid, and finally the pY-ads-8-5HRE-cfosp-IFNG eukaryotic plasmid was constructed. The constructed plasmid was identified by double enzyme digestion and gene sequencing (**Figure 1**).

### Preparation and characterization of Fe_3_O_4_ nanoparticles and PEI-Fe_3_O_4_ magnetic nanoparticles

Fe_3_O_4_ nanoparticles were synthetized by chemical coprecipitation method. The synthesis steps of Fe_3_O_4_ nanoparticles are shown below. A 100 ml solution of FeCl_3_ with a concentration of 0.1mol/L and a 600ml solution of FeCl_2_ with a concentration of 0.1mol/L were prepared. The two solutions were then added to a jar and mixed for 10 minutes under nitrogen protection. The PH of the mixed solution was adjusted to 9 with 1.5mol/L ammonia (22).

In the mixture, black sediment rapidly appeared. The suspension was stirred continuously for 30 minutes. After incubation at 90 degrees Celsius for 30 minutes, the jar was placed on a strong permanent magnet. When the black sediment had sunk to the bottom of the jar, the supernatant was discarded. The black sediment was washed three times with deionized water and dried under vacuum. The morphology, structure, and size of Fe_3_O_4_ nanoparticles were observed using a high-resolution transmission electron microscope (TEM) (JEM-2100, Jeol, Japan). The hydrated particle size and zeta potential of Fe_3_O_4_ were measured using a Zetasizer Nano (Nano ZS, Malvern, UK).

The modification of Fe_3_O_4_ nanoparticles with PEI was performed as follows (22): Fe_3_O_4_ magnetic fluid was prepared with buffered saline as a solvent at a 4% concentration. The magnetic fluid was dispersed with ultrasound as much as possible. The suspension was stirred on a shaking table at room temperature for 24 h. PEI-Fe_3_O_4_ nanoparticles were separated from the suspension using a permanent magnet and washed three times each with deionized water and methanol. The PEI-Fe_3_O_4_ nanoparticles were dried in a vacuum drying chamber.

The morphology and size of PEI-Fe_3_O_4_ nanoparticles were observed by high-resolution TEM. The hydrated particle size and zeta potential of PEI-Fe_3_O_4_ were measured by Zetasizer Nano. Fourier transform infrared spectroscopy (FTIR) (Nicolet IS5, Thermo, USA) was used to verify whether PEI was successfully modified on the surface of Fe_3_O_4_ nanoparticles.

### Preparation and characteristics of PEI-Fe_3_O_4_/pYr-ads-8-5HRE-cfosp-IFNG albumin nanospheres

In our previous experiments, we investigated the optimal mass ratio of PEI-Fe_3_O_4_ MNPs to plasmid pYr-ads-8-5HRE-cfosp-IFNG (30). Based on this mass ratio, a 20ml suspension was prepared and allowed to stand for 30 minutes. Then, 100 mg of BSA was added to the suspension stirred. The pH of the suspension was adjusted to 9 using NaOH. Ethanol was slowly dripped into the suspension at a rate of 1 mL/min, followed by the addition of 50 μL of 2.5% glutaraldehyde. The suspension was continuously stirred at room temperature for 24 h. After high-speed centrifugation, the supernatant was discarded, and the nanospheres were washed with deionized water three times to obtain PEI-Fe_3_O_4_/pYr-ads-8-5HRE-cfosp-IFNG albumin nanospheres. The nanospheres were characterized by TEM and Zetasizer Nano.

### The expression of pYr-ads-8-5HRE-cfosp-IFNG in HepG2 cells was detected by quantitative PCR

HepG2 cells were added to 6-well plates at a density of 3×10^5^ cells/well, and 2ml of cell suspension was added to each well. After 24 hours of incubation at 37°C, when the cell confluence level reached 80%, the cells were divided into 4 groups, and 2ml of following nanospheres and serum-free medium mixture was added to each group, respectively (1): PEI-Fe_3_O_4_ nanospheres. (2) PEI-Fe_3_O_4_/pDONR223-IFNG nanospheres. (3) PEI-Fe_3_O_4_/pYr-ads-8-cfosp-IFNG nanospheres. (4) PEI-Fe_3_O_4_/pYr-ads-8-5HRE-cfosp-IFNG nanospheres. Cells were incubated for 4 hours and fresh medium containing serum was added to the cells as a replacement. The cells were then incubated for 24 h at 37°C and exposed to 2Gy of X-ray (RS2000Pro, RAD, USA). The cells were further cultured for 48 h at 37°C under hypoxic conditions (1%O_2_, 5%CO_2_ and N_2_ in equilibrium). Total RNA was extracted from the cells after incubation. Untreated HepG2 cells served as a blank control group. The internal reference gene was GAPDH. Quantitative polymerase chain reaction (qPCR) was used to quantitatively analyzed the expression level of IFNγ gene. The primer sequences were obtained from Nan Jing Jin Sirui Company (China)

### Heating test of PEI-Fe_3_O_4_/pYr-ads-8-5HRE-cfosp-IFNG albumin nanospheres

The concentration of iron in the PEI-Fe_3_O_4_/pYr-ads-8-5HRE-cfosp-IFNG albumin nanospheres was determined using the thiocyanate spectrophotometric method (34). Various doses of PEI-Fe_3_O_4_/pYr-ads-8-5HRE-cfosp-IFNG albumin nanospheres were prepared as magnetic fluids with 5 mL normal saline. The iron concentrations in these magnetic fluids were 0.125, 0.25, 0.375, 0.5, 0.625, 0.75, 0.875, and 1.0 mg/mL, respectively. 2 mL of magnetic fluids with different concentrations were added into flat-bottomed vessels. These vessels were placed at the center of the hyperthermia coil of high-frequency AMF (alternating magnetic field) (SP-04C, Shuangping, China). The parameters of high-frequency electromagnetic field, including output power and output circuit, were set to 230kHz and 25A, respectively. The total heating time was 1 hour, and the temperature was measured every 5 minutes.

### The therapeutic effect of PEI-Fe_3_O_4_/pYr-ads-8-5HRE-cfosp-IFNG albumin nanospheres on hepatoma

#### CCK8 assay

HepG2 cells were cultured in three 96-well plates with a density of 4×10^4^ cells/well. The cells in the three plates were incubated for 24 h. Subsequently, the cells in each plate were divided into five groups as follows:

(1) The radiation-gene therapy and MFH combination group (PEI-Fe_3_O_4_/pYr-ads-8-5HRE-cfosp-IFNG albumin nanospheres were added);
(2) The radiation-gene therapy group (PEI-Fe_3_O_4_/pYr-ads-8-5HRE-cfosp-IFNG albumin nanospheres were added);
(3) The MFH group (PEI-Fe_3_O_4_/pYr-ads-8-5HRE-cfosp-IFNG albumin nanospheres were added);
(4) The radiation alone group (without nanospheres);
(5) Negative control group (without nanospheres).

PEI-Fe_3_O_4_/pYr-ads-8-5HRE-cfosp-IFNG albumin nanospheres were added to the MFH group, the radiation-gene therapy group, and the radiation-gene therapy group. An equal volume of DMEM nutrient solution was added to the radiation alone group and negative control group. The cells were incubated for another 24 hours at 37°C, and then the 96-well plates of the MFH group, the radiation-gene therapy group, and the radiation-gene therapy group were put on a strong permanent magnet (Magneto FACTOR plate) from Germany. All the groups that need to be heated were put on a high-frequency AMF and heated for 1 hour (f=230 kHz, I=25A) (35). All the radiation groups were exposed to 2Gy of X-ray radiation using an X-ray biological irradiator (30).

The three 96-well plates were incubated for 24 h,48 h, and 72 h, respectively. 10 μL of CCK8 solution was added into each well of the plates and incubated at 37 degrees Celsius for 1–4 h. The optical density (OD) of each group at 450 nm was measured by a spectrophotometer (Infinite 50, Tecan, CH). The relative inhibition rate (RIR) of cell proliferation (36) could be calculated using the following formula:


(1) $$\text{RIR}=(1-\frac{experimental\;group\;OD-blank\;control\;group\;OD}{negative\;control\;group\;OD-blank\;control\;group\;OD})\times 100\%$$

### The apoptosis rate of HepG2 cells was analyzed by flow cytometry

HepG2 cells were cultured in 5 culture vials with a density of 3×10^5^ cells/mL. Then, the grouping and treatment were the same as in the CCK8 experiment. At the end of the treatment, the cells were cultured for another 48 hours. The cells were resuspended with 1× binding buffer at a density of 1×10^6^ cells/mL after being washed with cold PBS three times. For each group, 100 μL of cell suspension was poured into a 5 mL tube. 5 μL of fluorescein isothiocyanate Annexin V and 5 μL of propidium iodide (PI) were added to each tube. After the cells were incubated for 15 min at 25°C in the dark, 400 μL of 1× binding buffer was added to each tube. The apoptosis of the cells was analyzed by flow cytometry within 1 hour. (FCM, Vantage SE, BD, USA) (37).

### Intracellular analysis of HepG2 cells treated with Fe_3_O_4_ nanoparticles by TEM

The intake of Fe_3_O_4_ nanoparticles in HepG2 cells was analyzed by TEM (JEM- 200CX, JBOL, Japan). HepG2 cells (4×10^4^) were grown in a 12-well culture plate for 24h and then incubated with Fe_3_O_4_ nanoparticles (PEI-Fe_3_O_4_/pYr-ads-8-cfosp-IFNG) under standard condition (37°C, 5% CO_2_). After incubation for 24 hours, the cells were fixed with 2.5% glutaraldehyde for more than 6 hours (4°C). Then, the cells were prepared according to the conventional procedure of transmission electron microscope ultrathin section. The finished samples were observed under TEM at 80 kV.

### Animal experiments

BALB/c nude mice (6-weeks-old, weighing 20–22 g), with half male and half female, were obtained from the Animal Experimental Center, Institute of Biochemistry and Cell Biology, Shanghai Academy for Biological Sciences, China. The animal experiments were approved by Ethics Committee of North Sichuan Medical College (NSMC-AEC 2021 [157]) and carried out according to institutional guidelines. The nude mice were raised in the Experimental Center of North Sichuan Medical College. HepG2 cells at a density of 2 ×10^6^ cells were injected subcutaneously into the buttocks of the right hind limbs of the mice.

When the transplanted tumor grew to 1 cm in diameter, the mice were divided into five groups, each containing six mice. A multi-point injection strategy was used to inject the required drugs at 3, 6, 9 and 12 points of the transplanted tumor. The specific procedures were as follows:

(1) Radiation-gene therapy and MFH combination group: PEI-Fe_3_O_4_/pYr-ads-8-5HRE-cfosp-IFNG albumin nanospheres (plasmid: 10 μg/mouse), combined with high-frequency AMF exposure.
(2) The radiation-gene therapy group: PEI-Fe_3_O_4_/pYr-ads-8-5HRE-cfosp-IFNG albumin nanospheres (plaimid: 10 μg/mouse).
(3) MFH group: PEI-Fe_3_O_4_/pYr-ads-8-5HRE-cfosp-IFNG albumin nanospheres (plasmid: 10 μg/mouse), heated on a high frequency AMF for 1 hour.
(4) Radiation alone group:1 ml PEI-Fe_3_O_4_ albumin nanospheres;
(5) Negative control group:1 ml 0.9% NaCl.

The tumors in groups (1), (3), and (4) received 2 Gy of X-ray radiation under an X-ray biological irradiator every other day, three times in total. The tumors in groups (1) and (3) were heated on a high frequency AMF for 1 hour, with a frequency of 230 kHz and a current of 25A. The temperature of the tumors was measured at multiple points using an infrared thermometer (ZyTemp-TN18, Xingtaiheng, China). After treatment, the nude mice were fed for 6 weeks and then sacrificed. The volume and mass of the tumors were measured. And the tumor mass and volume inhibition rate (38) were calculated according to the following formula.


(2) $$\text{Mass inhibition}=(1-\frac{mean\;tumor\;mass\;of\;the\;experimental\;group}{mean\;tumor\;mass\;of\;the\;control\;goup})\times 100\%$$

(3) $$\text{Volume inhibition}=(1-\frac{mean\;tumor\;volume\;of\;the\;experimental\;group}{mean\;tumor\;volume\;of\;the\;control\;group})\times 100\%$$

### Long-term safety of Fe_3_O_4_ magnetic nanoparticles

Before sacrificing the mice, blood samples were collected in EDTA-coated tubes, and hematological parameters were analyzed immediately using an automatic hematology analyzer (KX-21, Sysmex, Japan), including platelet (PLT), red blood cell (RBC), white blood cell (WBC), and hemoglobin (HGB). Additionally, an automated analyzer platform (Cobas C501, Roche, Chian) was used to measure creatinine (CREA), alkaline phosphatase (ALP), triglycerides (TGs), total cholesterol (TC), high-density lipoprotein (HDL), low-density lipoprotein (LDL), and blood urea nitrogen (UREA). Histopathological evaluations were performed to assess any potential adverse effects of the treatments. After the experiment, the heart, spleen, lung, and kidney of the nude mice were removed and fixed with paraformaldehyde to prepare pathological sections for observation under the microscope.

### Statistical analysis

All experiment data were processed by SPSS 25.0 software. *P*<0.05 was considered statistically significant.

## Results

### Identification of pYr-ads-8-5HRE-cfosp-IFNG

**Figure 2:**
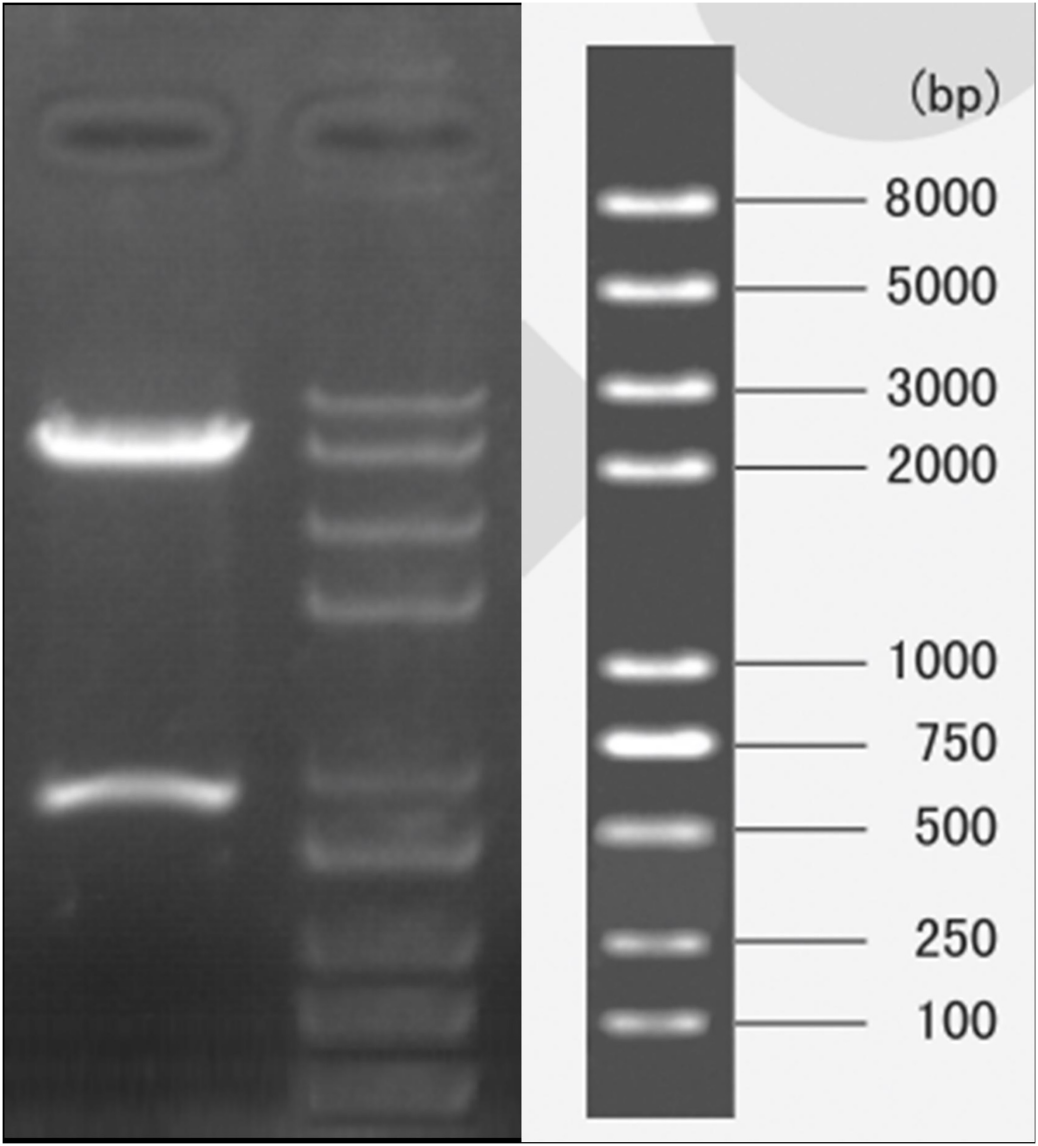
Gel electrophoresis of pYr-5HRE-cfosp-IFNG plasmid after double enzyme digested. Lane M, marker; lane 1, pYr-5HRE -cfosp-IFNG plasmid.

Because the theoretical structure of p5HRE-cfosp-IFNG is clear, double digestion of the constructed plasmid with EcoRI and MluI restriction enzymes will result in 800 bp fragment and a 5.5 K fragment. Agarose gel electrophoresis verified that the obtained fragments from the digested plasmid were consistent with the predicted outcomes (**Figure 2**).

**Figure 3:**
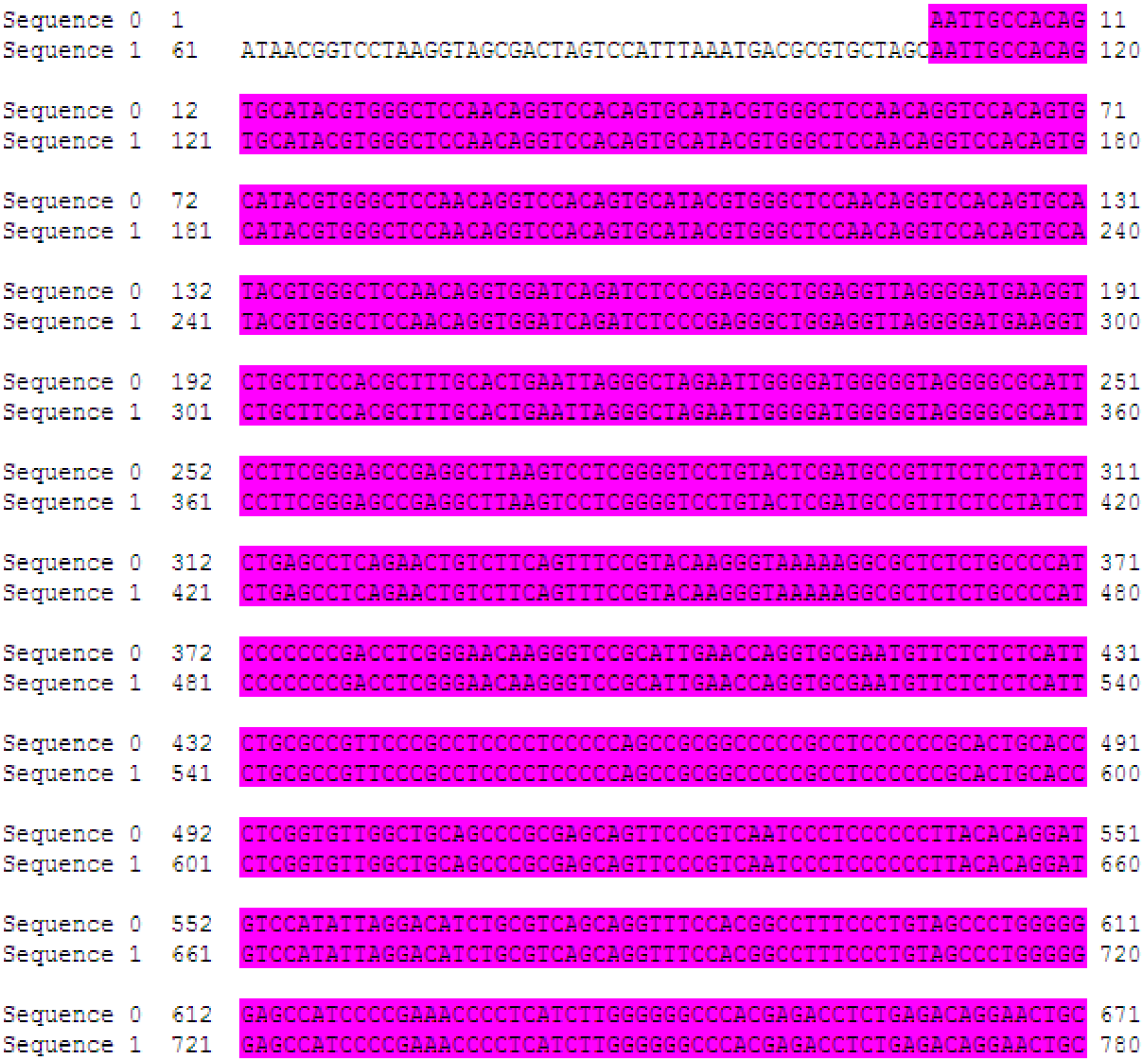
The sequencing results of pYr-5HRE-cfosp-IFNG. Sequence 0, template sequence; sequence 1, pYr-5HRE-cfosp-IFNG. Comparison software used was Dnassit 2.0 (Dnassit, Imola, BO, Italy).

Furthermore, the sequencing results of the self-constructed pYr-5HRE-cfosp-IFNG plasmid were compared to the reference sequence, and the findings are presented in **Figure 3**. The complete sequence was accurate, indicating successfully synthesis of the pYr-5HRE-cfosp-IFNG plasmid.

### Preparation and characterization of the Fe_3_O_4_ nanoparticles and PEI -Fe_3_O_4_ nanoparticles

**Figure 4:**
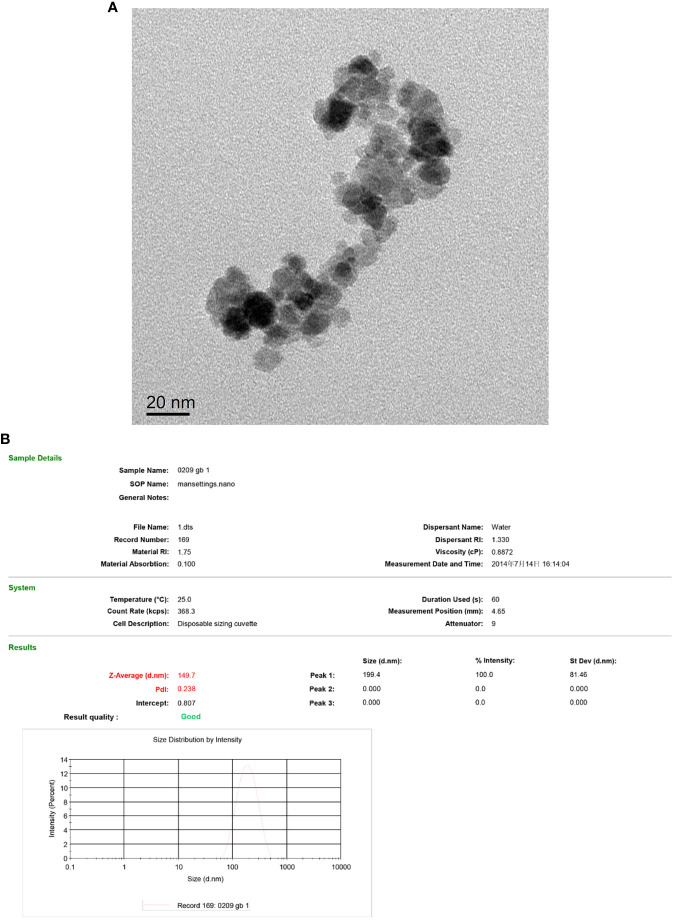
TEM of the Fe_3_O_4_ nanoparticles **(A)** and hydrodynamic diameter distribution of Fe_3_O_4_ nanoparticles **(B)**.

According to transmission electron microscopy, the Fe_3_O_4_ nanoparticles display a high electron density with a diameter of approximately 10 nm. (**Figure 4A**) The hydrodynamic size of Fe_3_O_4_ nanoparticles was 149.7 nm with a polydispersity index (PdI) of 0.238 (**Figure 4B**).

**Figure 5:**
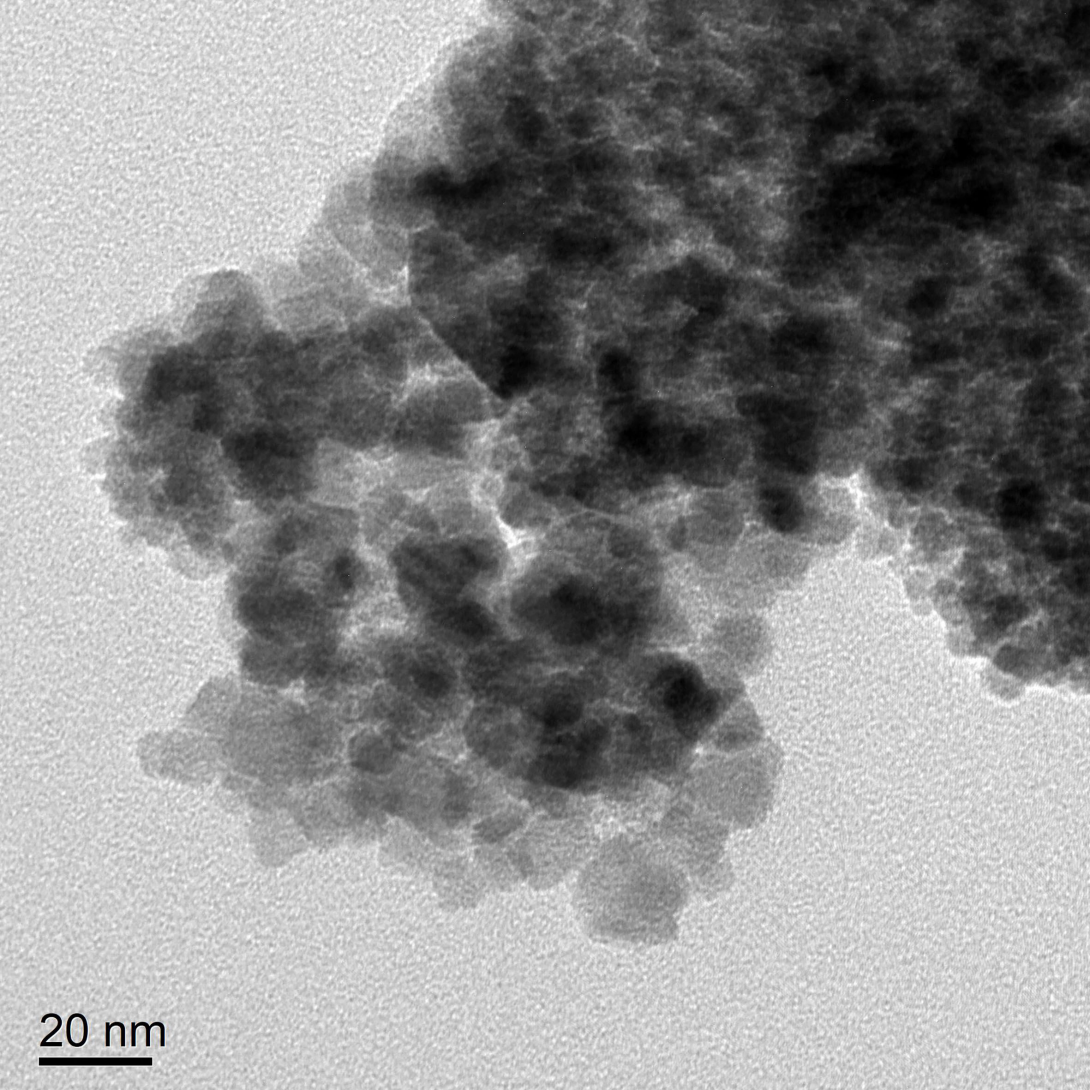
TEM of the PEI -Fe_3_O_4_ nanoparticles.

**Figure 6:**
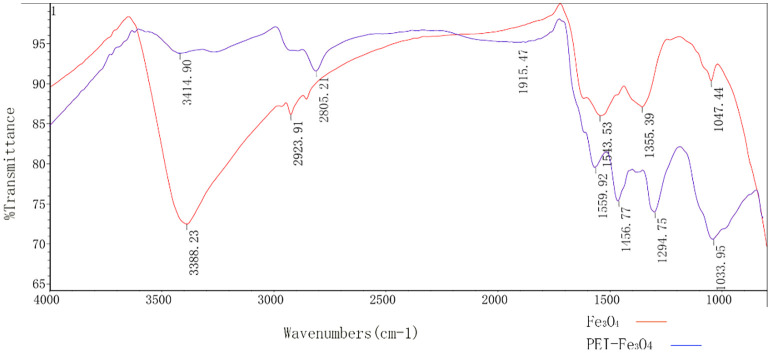
FTIR spectra of Fe_3_O_4_ nanoparticles and PEI -Fe_3_O_4_ nanoparticles.

**Figure 7:**
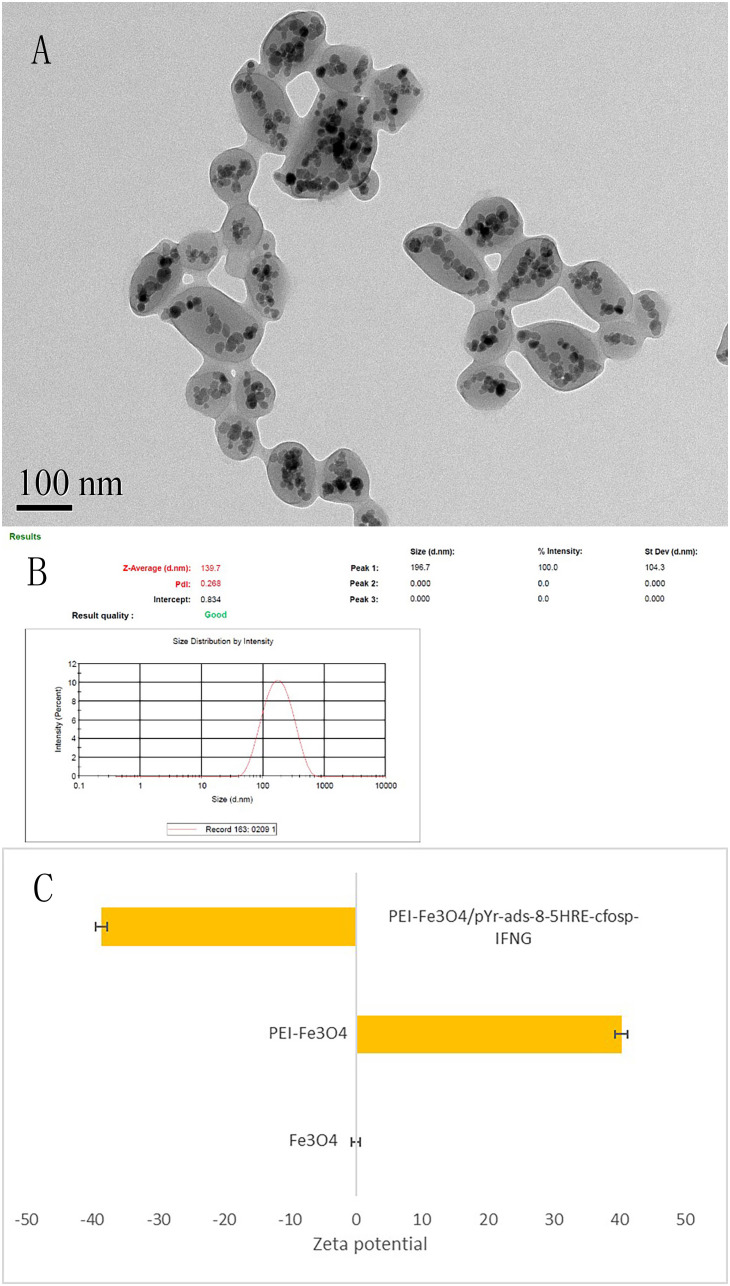
Characterization of PEI-Fe_3_O_4_/pYr-ads-8-5HRE-cfosp-IFNG albumin nanospheres. **(A)** TEM of PEI-Fe_3_O_4_/pYr-ads-8-5HRE-cfosp-IFNG albumin nanospheres. **(B)** Hydrodynamic diameter distribution of PEI-Fe_3_O_4_/pYr-ads-8-5HRE-cfosp-IFNG albumin nanospheres. **(C)** The zeta potential of Fe_3_O_4,_ PEI-Fe_3_O_4_ and PEI-Fe_3_O_4_/pYr-ads-8-5HRE-cfosp-IFNG albumin nanospheres in pH7.

The size of PEI-Fe_3_O_4_ nanoparticles under TEM was found to be similar to that of Fe_3_O_4_, which a diameter of approximately 10 nm (**Figure 5**). Furthermore, FTIR results revealed the presence of specific peaks at 3,414.9 cm-1, 2,805.2cm-1 and 1,456.8 cm-1, which is consistent with the unique chemical structure of PEI (**Figure 6**). The zeta potential test results indicated that the surface charge of Fe_3_O_4_ nanoparticles at pH 7 was 0 ± 0.7 mV, while the surface charge of PEI - Fe_3_O_4_ nanoparticles increased to 40.3 ± 1 mV (**Figure 7C**).

### Preparation and characteristics of PEI-Fe_3_O_4_/pYr-ads-8-5HRE-cfosp-IFNG albumin nanospheres

TEM revealed that the self-prepared PEI-Fe_3_O_4_/pYr-ads-8-5HRE-cfosp-IFNG albumin nanospheres exhibit an approximately spherical or elliptical shape. The albumin nanospheres were utilized as encapsulating agents for Fe_3_O_4_ nanoparticles with high electron density (**Figure 7A**). The hydrodynamic size of the albumin nanospheres was 139.7 nm with a polydispersity index (PdI) of 0.268 (**Figure 7B**). Zeta potential test results indicated that the surface charge of the magnetic albumin nanospheres was -38.6 ± 0.9 mV.

### The transcript of pYr-ads-8-5HRE-cfosp-IFNG in HepG2 cells was detected by quantitative PCR

**Figure 8:**
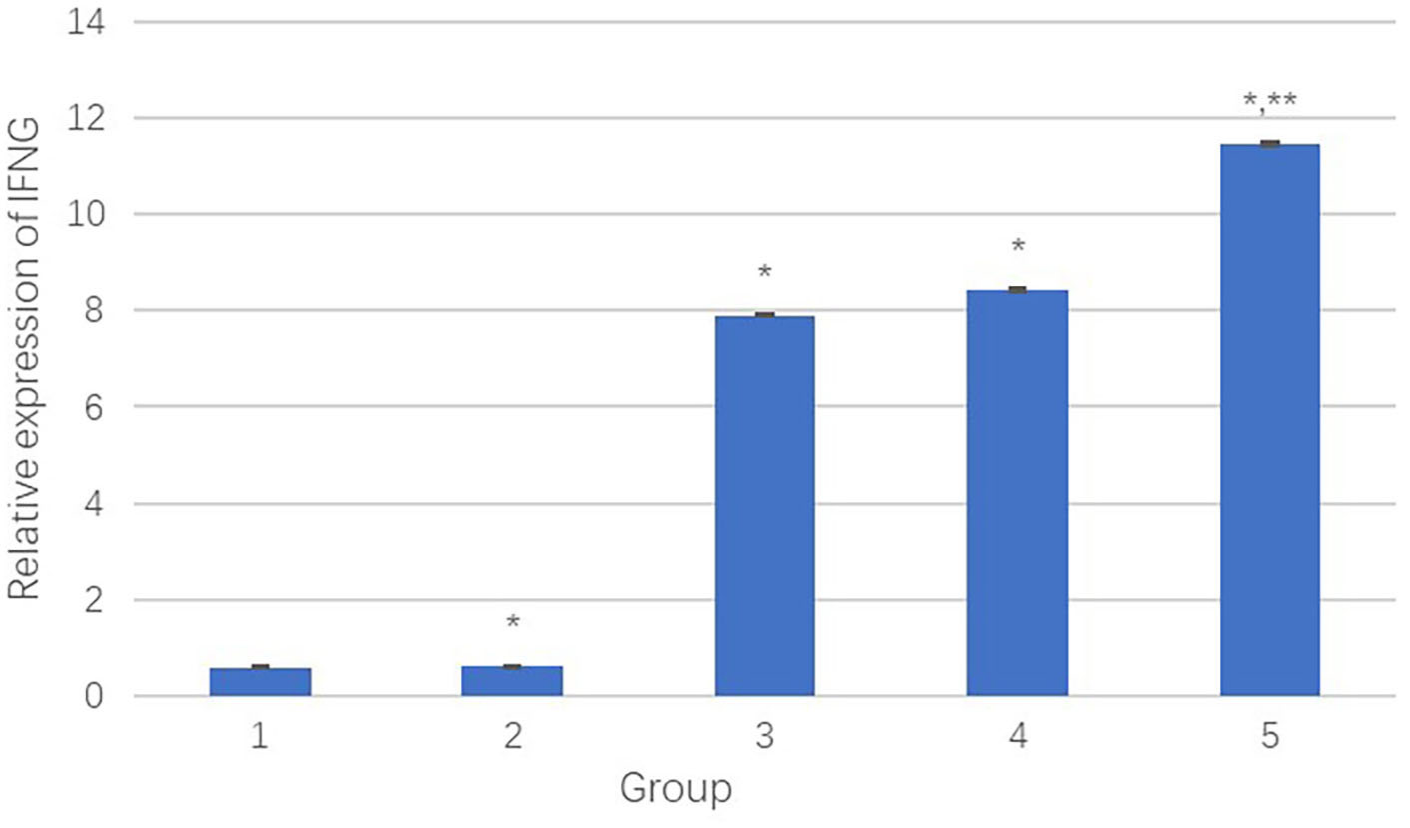
The relative transcript quantity of IFNγ. *Comparison between the experimental group and the negative control group, *P*<0.05; **Comparison between group 4 and group 5, *P*<0.05.

The IFNG mRNA levels in HepG2 cells were detected by qPCR. **Figure 8** presents the relative transcript levels of IFNG in each group.

(1) The negative control group;
(2) The PEI-Fe_3_O_4_ albumin nanospheres group;
(3) The PEI-Fe_3_O_4_/pDONR223-IFNG albumin nanospheres group;
(4) The PEI-Fe_3_O_4_/pYr-ads-8-cfosp-IFNG albumin nanospheres group;
(5) The PEI-Fe_3_O_4_/pYr-ads-8-5HRE-cfosp-IFNG albumin nanospheres group.

Results showed that compared to the negative control group, the PEI-Fe_3_O_4_/pDONR223-IFNG group, PEI-Fe_3_O_4_/pYr-ads-8-cfosp-IFNG group, and PEI-Fe_3_O_4_/pYr-ads-8-5HRE-cfosp-IFNG group had significantly higher IFNγ transcript, while the PEI-Fe_3_O_4_ group exhibited few IFNγ transcript. Moreover, the IFNγ transcript level in the PEI-Fe_3_O_4_/pYr-ads-8-5HRE-cfosp-IFNG group was higher than that in the PEI-Fe_3_O_4_/pYr-ads-8-cfosp-IFNG group after being treated with hypoxia (*P*<0.05).

### Heating test of PEI-Fe_3_O_4_/pYr-ads-8-5HRE-cfosp-IFNG albumin nanospheres

**Figure 9:**
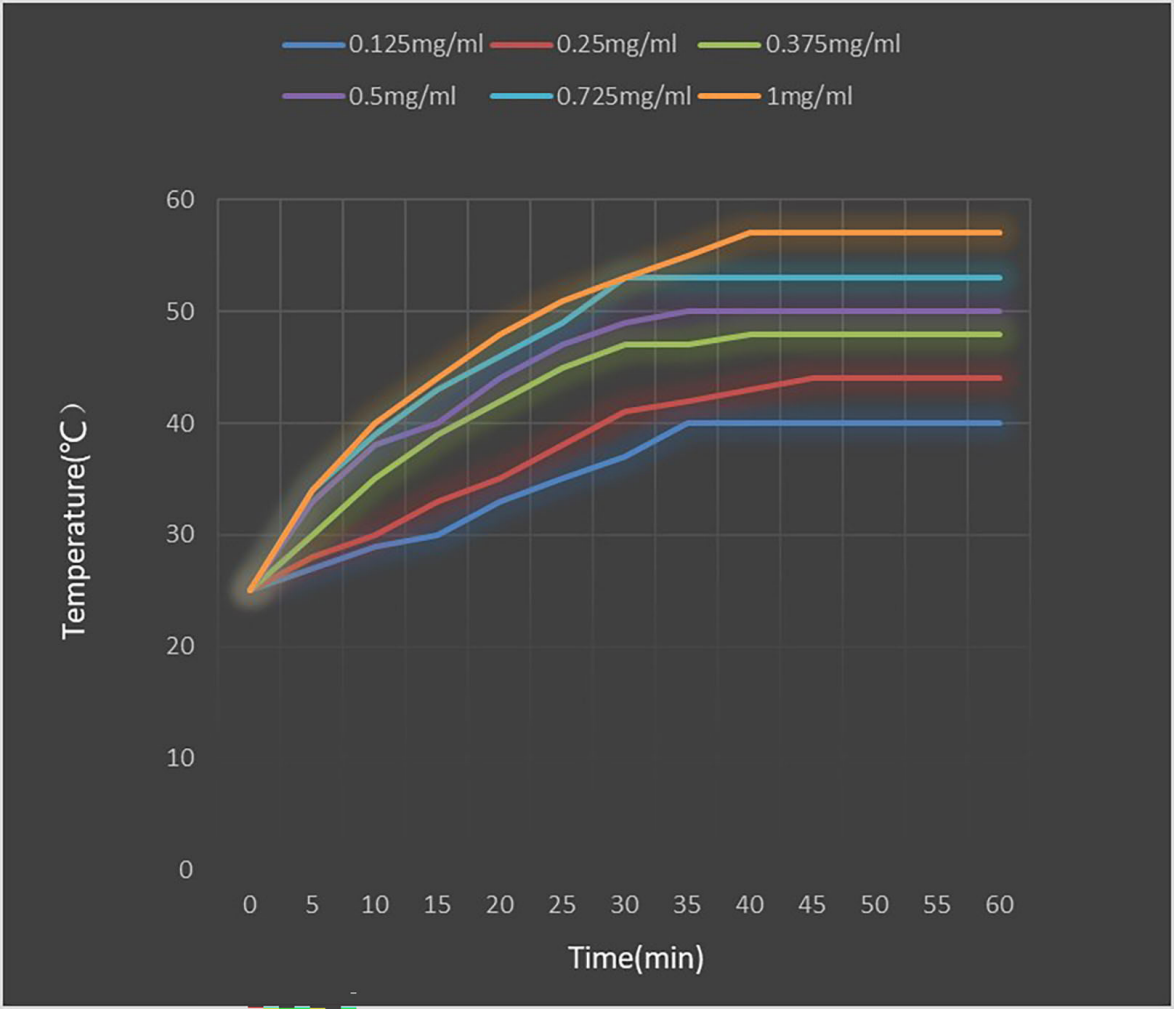
Heating test of PEI-Fe_3_O_4_/pYr-ads-8-5HRE-cfosp-IFNG albumin nanospheres.

**Figure 9** shows the temperature rise curve of the PEI-Fe_3_O_4_/pYr-ads-8-5HRE-cfosp-IFNG albumin nanospheres at different concentrations. Upon exposed to an alternating magnetic field, the temperature of all concentrations of fluid increases quickly, and remains constant at a certain temperature. The maximum temperature that can be reached is higher for higher concentrations of magnetic fluid. However, the heating rate of solutions with different concentrations is not significantly different. Within the first 5 minutes of heating, the magnetic fluid rapidly heat up, reaching the highest temperature in about 35 minutes, and then maintaining at this temperature. The maximum temperature of 0.25 mg/mL solution is stable at about 44°C, which is suitable for tumor thermal therapy without damaging normal tissues. Therefore, a concentration of 0.25 mg/mL was deemed suitable for the subsequent MFH experiment.

### The therapeutic effect of PEI-Fe_3_O_4_/pYr-ads-8-5HRE-cfosp-IFNG albumin nanospheres on hepatoma

#### CCK8 assay

**Figure 10:**
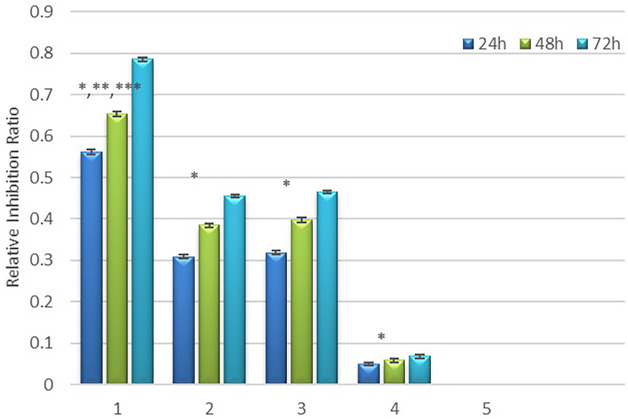
Relative inhibition rate in each group. * Comparison between the experimental group and the negative control group, *P*<0.05; ** Compared to group 4, *P*<0.05, *** Compared to group 2 and 3, *P*<0.05.

**Figure 10** presents the results of the relative inhibition rate of cell proliferation. The difference in relative inhibition rate between the experimental groups and the negative control group was statistically significant. (*P*<0.05). **Table 1** and **Figure 10** show the relative inhibition rate of each group at 24h, 48h, and 72h. It can be observed that the combination treatment (Group 1) exhibited the highest relative inhibition rate. Radiation-alone slightly inhibited the growth of HepG2 cells. Both MFH and radiation-gene therapy can significantly restrain the growth of HepG2 cells, but the relative inhibition rate was still lower than that of the combined therapy.

**Table 1:** Relative inhibition rate (RIR) in each group.

Group	Relative inhibition rate (RIR)%		
	24 h	48 h	72 h
1. The radiation-gene therapy and MFH combination group	56.24	65.34	78.50
2. The radiation-gene therapy group	30.92	38.48	45.49
3. The MFH group	31.87	39.76	46.50
4. The radiation alone group	4.93	5.85	6.82
5. The negative control group	0	0	0

### Flow cytometry assay

**Figure 11:**
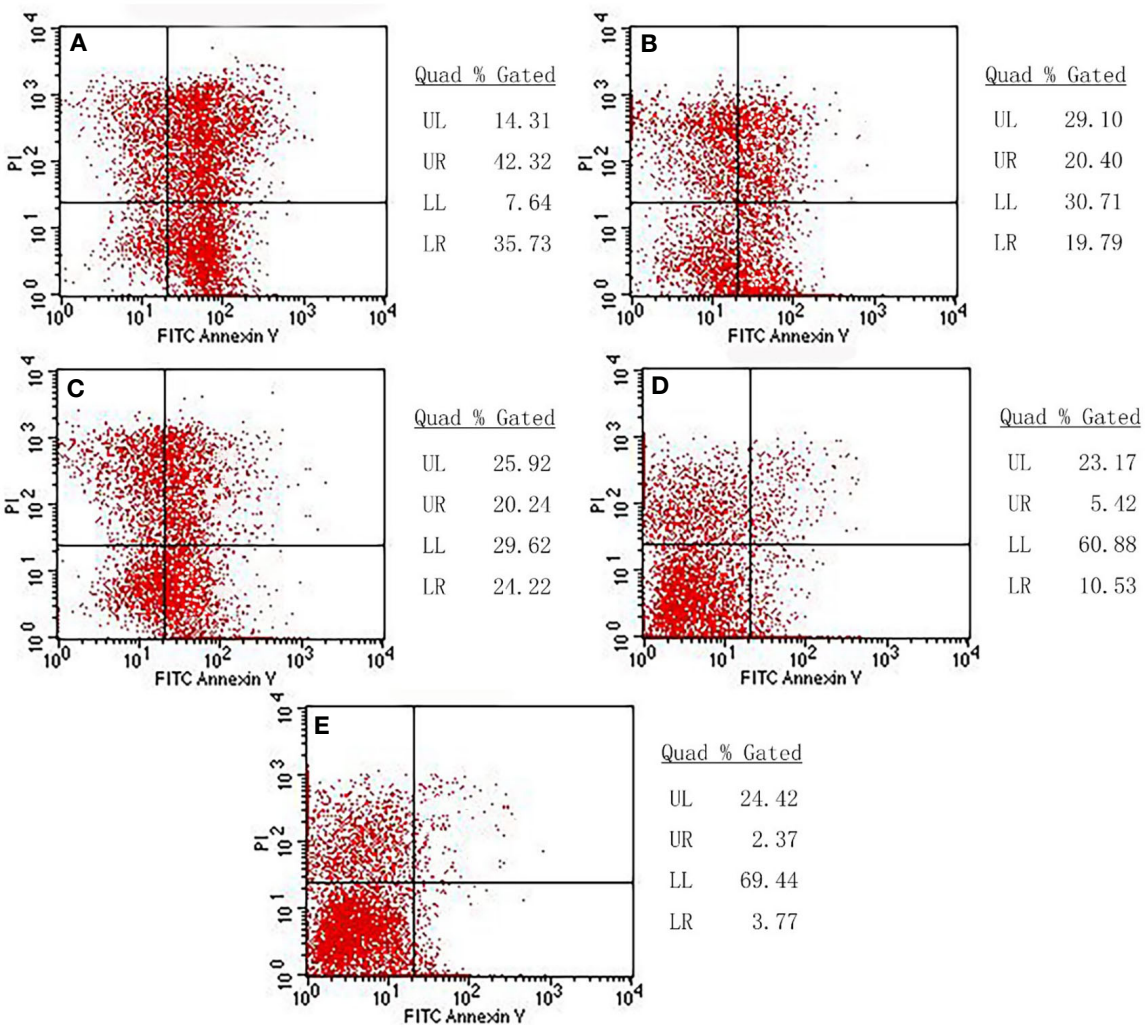
The apoptotic rate and necrotic rate of each group. **(A)** Radiation-gene therapy and MFH combination group **(B)** Radiation-gene therapy group; **(C)** MFH group; **(D)** Radiation alone group; **(E)** Negative control group.

Flow cytometry was employed to analyze whether cell death was due to necrosis or apoptosis. Our results showed different degrees of apoptosis and necrosis in each experimental group (**Figure 11**). In the combination treatment group, the apoptotic rate and necrotic rate were 42.32% and 35.73%, respectively, indicating that the cell death was mainly due to apoptosis. The apoptotic rate and necrotic rate in the radiation-gene therapy group were 20.40% and19.79%, respectively. The MFH group had apoptotic and necrotic of 20.24% and 24.22%, respectively. The radiation alone group had the lowest rates of apoptotic rate and necrotic cells, at 5.42% and 10.53%, respectively. The combination of radiation-gene therapy and MFH exhibited better efficacy than any other group.

### Intracellular analysis of HepG2 cells treated with Fe_3_O_4_ nanoparticles by TEM

**Figure 12:**
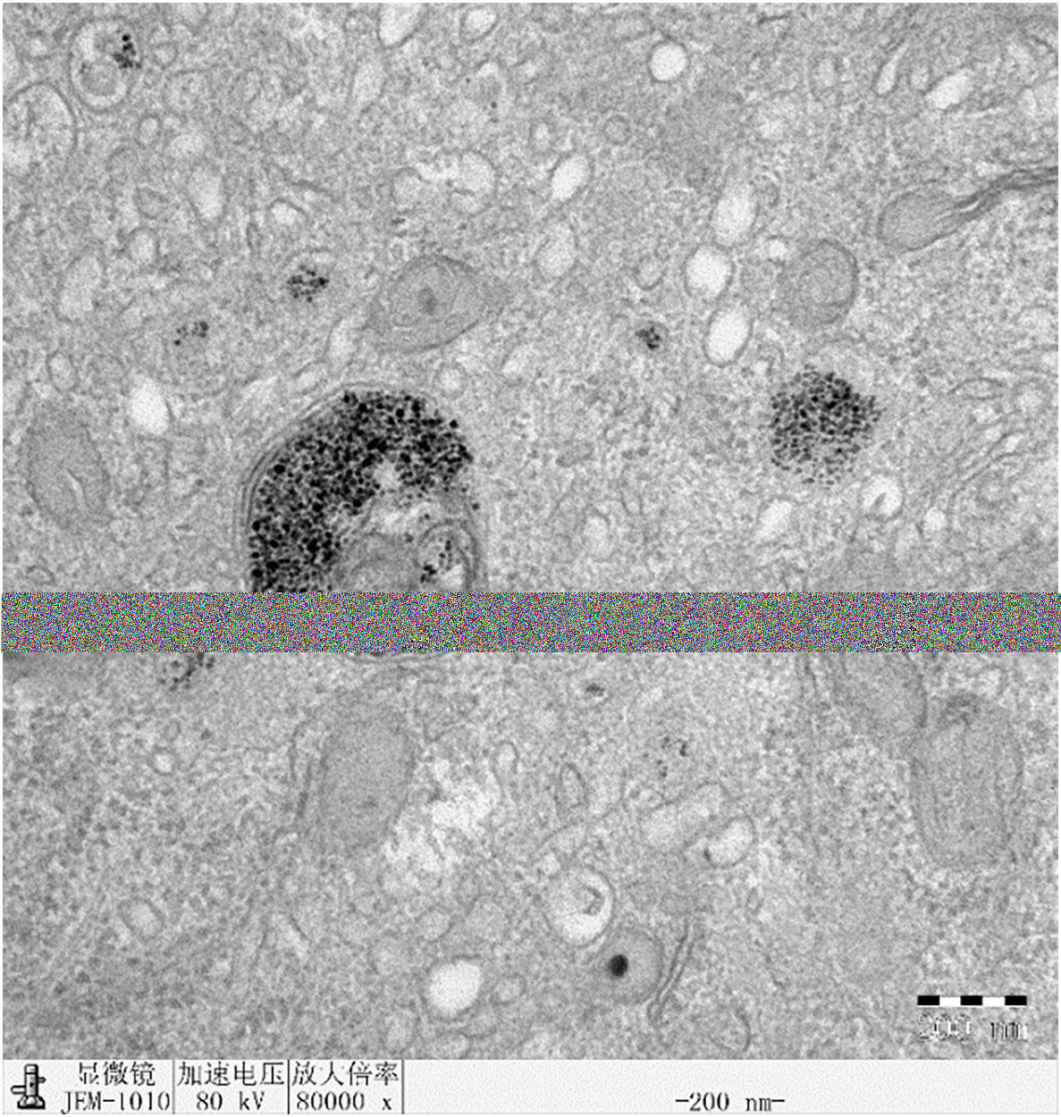
TEM of HepG2 cell containing Fe_3_O_4_ nanoparticles.

Fe_3_O_4_ nanoparticles successfully internalized by HepG2 cells, as shown in **Figure 12**. The majority of the nanoparticles were localized in lysosomes.

### Animal experiments (*in vivo* experiments of combination therapy)

The tumor mass and volume inhibition rates of the radiation-gene therapy and MFH combination group were 66.67% and72.53%, respectively. which were significantly higher than those of the other groups (**Table 2**). The combination treatment of radiation-gene therapy and MFH showed a significant inhibitory effect on tumor growth. Radiation alone, MFH alone, and radiation-gene therapy alone also showed inhibitory effects on tumor growth, but the inhibition rates were weaker than those of the combination treatment group. However, there was no significant change in the tumor mass and volume inhibition rate in the radiation alone group, which was not statistically significant when compared with the negative control group.

**Table 2:** Mass and volume inhibition of transplanted tumor of hepatoma in nude mice.

Group	Tumor mass (g) ( $\bar{\text{X}}$ ± S, n=6)	Mass inhibition (%)	Tumor volume (mm^3^) ( $\bar{\text{X}}$ ± S, n=6)	Volume inhibition (%)
The radiation-gene therapy and MFH combination group	0.38 ± 0.021	66.67^abcd^	335.31 ± 128.09	72.53^abcd^
The radiation-gene therapy group	0.76 ± 0.023	33.33^a^	756.32 ± 123.43	38.03^a^
The MFH group	0.72 ± 0.013	36.84^a^	724.76 ± 130.21	40.63^a^
The radiation alone group	1.08 ± 0.015	5.26^a^	1180.00 ± 120.86	3.33^a^
Negative control group	1.14 ± 0.020	–	1220.65 ± 113.50	–

^a^ *P*<0.05 compared to the negative control group. ^b^ *P*<0.05 compared to radiation-gene therapy group. ^c^ *P*<0.05 compared to MFH group. ^d^ *P*<0.05 compared to radiation alone group.

$\bar{\text{X}}$ ± S: Mean ± standard deviation.

### Long-term safety of Fe_3_O_4_ magnetic nanoparticles

**Figure 13:**
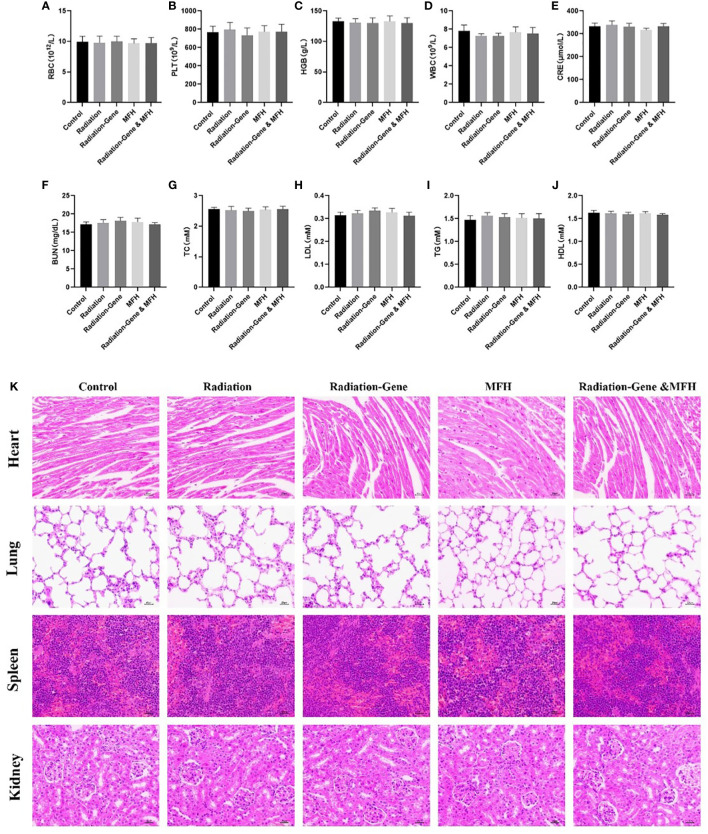
Long term safety of nanoparticle after treatment with different formulations. **(A–J)** RBC, PLT, HGB, WBC, CRE, BUN, TC, LDL, TG, HDL. **(K)** Histological evaluation of organs (heart, lung, spleen and kidney) from HCC mice two months post treatments.

During drug treatment, the potential toxic side effects of anti-tumor nanoparticles are important considerations for further clinical application. To assess the biosafety of our treatment, six weeks after treatment. The mice were investigated for potential side effects before they were sacrificed. Clinical biochemistry analysis, including platelet (PLT), red blood cell (RBC), white blood cell (WBC), hemoglobin (HGB), creatinine (CREA), alkaline phosphatase (ALP), triglycerides (TGs), total cholesterol (TC), high-density lipoprotein (HDL), low-density lipoprotein (LDL) and blood urea nitrogen (UREA), were performed (**Figure 13**). The results showed that these indexes did not significantly change during the treatment, indicating that the liver and kidney functions of nude mice were not impaired by the treatment. Moreover, no obvious pathological injury or inflammatory lesions were found in the tissue sections of the heart, lung, spleen and kidney of the five groups of nude mice, further indicating the biosafety of Fe_3_O_4_ magnetic nanoparticles.

## Discussion

Cancer remains one of the most severe diseases facing humankind. As far as current medical knowledge goes, there is no single treatment capable of curing cancer. Recent studies have shown that multi-mode therapy is often more effective than single-mode therapy in inhibiting tumors (39–41). In our study, we investigated the combined treatment of immunogene therapy and magnetic fluid hyperthermia using Fe_3_O_4_ nanoparticles as the medium on cancer, which demonstrated strong synergies.

Hyperthermia is a common adjuvant treatment for cancer and is used in various cancers. Increasing the temperature of tumor tissue to 40-43 degrees Celsius can effectively induce tumor cell death (42). The hyperthermia of tumor with nano-magnetic fluid involves directly inject MNPs into malignant tumor and heating them in AMF. This method has the advantages of accuracy, targeting, and temperature control and avoids overheating the normal tissues around the tumor (43). To ensure that the liquid is dispersed in the tumor tissues and prevent overflow of the liquid, we adopted a multi-point injection strategy, injecting the magnetic nano-composite materials into the 3rd, 6th, 9th, and 12th points of each tumor. Tumors are more sensitive to heating than normal tissues because of their unique internal microenvironment, such as hypoxia, vascular disorder, acidosis, and hypoperfusion (44). Therefore, hyperthermia is a promising treatment for tumors (45, 46). In our research, the tumor inhibition rate of the MFH group was the second highest, confirming the great potential of MFH in the treatment of solid tumors.

In our study, the tumor inhibition rate was significantly increased when MFH was combined with immunogene therapy. However, the mechanism underlying the synergistic effect of the combination therapy has not been investigated in this study. The following possibilities are speculated: hyperthermia in combination therapy of tumors was considered to influence the susceptibility to other manipulations mostly through microenvironmental factors (47). High temperature can destroy the biological integrity of the cell membrane and increase its permeability, which is conducive to the penetration and absorption of chemical drugs and gene transfer. The interior of solid tumors is often anoxic due to vascular disorder, while the anoxic cells are usually acidic and nutrient-poor, making them more vulnerable to heat damage. However, high temperature can temporarily improve the oxygen and blood supply to the tumor area, helping drugs to enter the tumor and improving the radiation susceptivity of tumor cells (48). Our next research goal is to explore the molecular mechanism of hyperthermia combined with immunogene therapy for hepatoma and to elucidate why combined therapy is superior to single therapy. This will guide the clinical selection of a more targeted and individualized treatment plan.

MNPs can not only be used as the medium of magnetic hyperthermia, but also as gene carriers in this study. Nanomaterials are used in many areas of biomedicine, including drug/gene therapy, diagnostic imaging (as contrast agents), and biological scaffolds. This is due to the advantages of nanomaterials such as high biosafety, complex surface functionalization, and easy preparation (49). MNPs have been used as gene vectors due to their good biocompatibility and large specific surface area, especially their ability to magnetically target to target tissues, which plays an important role in gene transfer (50, 51). Since DNA carries a negative charge, we connect PEI on the surface of Fe_3_O_4_-MNPs. PEI carries a positive charge and can bind to negatively charged DNA molecules. Thus, the plasmid can be effectively carried into HepG2 cells. **Figure 6** shows specific peaks at 3,414.9 cm-1, 2,805.2cm-1 and 1,456.8 cm-1, which are consistent with the special chemical structure of PEI. This indicates that PEI has successfully modified the surface of nano Fe_3_O_4_. Magnetic nanoparticles are easy to agglomerate because of their small particle size. Therefore, in this study, the particle size of MNPs measured by TEM is smaller than that detected by Zetasizer Nano. The Zetasizer Nano measurement shows the hydrodynamic size of the MNPs. However, when we coated the magnetic nanoparticles with albumin, the diameter of the nanoparticles observed by TEM was not very different from that measured by DLS. MNPs modified with albumin have better dispersion. More importantly, albumin is a substance already in the human body. Wrapping magnetic nanomaterials in albumin can improve the safety of nanomaterials and effectively avoid the removal of foreign substances by the body (52, 53).

Using nano-magnetic materials as a carrier, we combined MFH and radiation-induced immunogene therapy to explore a new therapy model for hepatoma. In our research, in addition to the combined treatment group, an experimental group was also set up for each single treatment method for comparison. The consequences of our study showed that the tumor inhibition rate of combination therapy was better than that of single therapy in both cell and animal experiments. As mentioned above, we still have a lot of work to do on the molecular mechanisms. Understanding the deeper issues is helpful in guiding the formulation and selection of clinical treatment. However, this study still has high reference value and research significance in exploring the comprehensive treatment of hepatoma.

## Conclusion

The results presented in this study demonstrate the successful synthesis and preparation of PEI-Fe_3_O_4_/pYr-ads-8-5HRE-cfosp-IFNG albumin nanospheres for targeted delivery of IFNG to hepatoma cells. The identification of pYr-ads-8-5HRE-cfosp-IFNG confirmed the successful synthesis of the plasmid. The characterization of Fe_3_O_4_ nanoparticles and PEI-Fe_3_O_4_ nanoparticles indicated their appropriate size and surface charge for subsequent experiments. The transcript levels of IFNG in HepG2 cells were detected by qPCR, and the results showed that the IFNG transcript levels in the PEI-Fe_3_O_4_/pYr-ads-8-5HRE-cfosp-IFNG group were higher than those in the PEI-Fe_3_O_4_/pYr-ads-8-cfosp-IFNG group after hypoxia treatment, suggesting that the addition of the HRE promoter enhanced the therapeutic effect of IFNG in hypoxic conditions. The results of *in vitro* and *in vivo* experiments showed that the relative inhibition rate of cell proliferation was significantly higher in the PEI-Fe_3_O_4_/pYr-ads-8-5HRE-cfosp-IFNG albumin nanospheres group compared to the other groups, including the negative control group and the PEI-Fe_3_O_4_/pDONR223-IFNG group. The results suggested that the targeted delivery of IFNG by the PEI-Fe_3_O_4_/pYr-ads-8-5HRE-cfosp-IFNG albumin nanospheres was effective in inhibiting the proliferation of hepatoma cells. These findings suggest the potential of using targeted delivery of IFNG by nanocarriers for the treatment of hepatoma.

## Data availability statement

The original contributions presented in the study are included in the article/supplementary material. Further inquiries can be directed to the corresponding author.

## Ethics statement

The animal study was reviewed and approved by Ethics Committee of North Sichuan Medical College.

## Author contributions

HZ: performed experiments, analyzed data, interpreted results of experiments, drafted manuscript, edited and revised manuscript; SL: analyzed data, approved final version of manuscript; FC: performed experiments, analyzed data; XM: approved final version of manuscript, analyzed data; ML: conceived and designed research. All authors contributed to the article and approved the submitted version.
